# Barriers and supports to implementation of MDI/spacer use in nine Canadian pediatric emergency departments: a qualitative study

**DOI:** 10.1186/1748-5908-4-65

**Published:** 2009-10-13

**Authors:** Shannon D Scott, Martin H Osmond, Kathy A O'Leary, Ian D Graham, Jeremy Grimshaw, Terry Klassen, the Pediatric Emergency Research Canada (PERC) MDI/spacer Study Group

**Affiliations:** 1 Faculty of Nursing, Clinical Sciences Building, University of Alberta, Edmonton, Alberta, Canada; 2 Department of Pediatrics, University of Ottawa, Ontario, Canada; 3 Department of Pediatrics, University of Alberta, Edmonton, Alberta, Canada; 4 School of Nursing and Department of Epidemiology and Community Medicine, University of Ottawa, Ontario, Canada; 5 Knowledge Translation Portfolio, Canadian Institutes of Health Research, Ottawa, Ontario; 6 Clinical Epidemiology Program, Ottawa Health Research Institute, Ottawa, Ontario, Canada; 7 Department of Medicine, University of Ottawa, Ontario, Canada

## Abstract

**Background:**

Despite recent research supporting the use of metered dose inhalers with spacer devices (MDI/spacers) in pediatric emergency departments (PEDs) for acute exacerbations of asthma, uptake of this practice has been slow. The objectives of this study were to determine the barriers and supports to implementing MDI/spacer research and to identify factors associated with early and late adoption of MDI/spacers in Canadian PEDs.

**Methods:**

Using a comparative case study design, we classified nine tertiary care pediatric hospital PEDs based on their stage of implementation. Data were collected using focus group interviews with physicians, registered nurses (RNs), and respiratory therapists (RTs), and individual interviews with both patient care and medical directors at each site. Initial coding was based on the Ottawa Model of Research Use (OMRU) categories of elements known to influence the uptake of innovations.

**Results:**

One hundred and fifty healthcare professionals from nine different healthcare institutions participated in this study. Lack of leadership in the form of a research champion, a lack of consensus about the benefits of MDI/spacers among staff, perceived resistance from patients/parents, and perceived increased cost and workload associated with MDI/spacer use were the most prevalent barriers to the adoption of the MDI/spacer. Common strategies used by early-adopting sites included the active participation of all professional groups in the adoption process in addition to a well-planned and executed educational component for staff, patients, and families. Early adopter sites were also more likely to have the MDI/spacer included in a clinical protocol/pathway.

**Conclusion:**

Potential barriers and supports to implementation have been identified that will help EDs adopt MDI/spacer use. Future interventions intended to increase MDI/spacer use in PEDs will need to be sensitive to the barriers identified in this study.

## Background

An acute asthma exacerbation is one of the most common reasons for children to present to an emergency department (ED). Conventional treatment focuses on the delivery of beta-2-agonists (bronchodilators) to relieve the bronchospasm. There are two main methods of delivering bronchodilators to children in the ED: nebulisation and metered-dose inhaler with spacer (MDI/spacer). For over a decade, the evidence has been well established that MDI/spacers are as effective (and in many ways superior) to nebulisers for mild to moderate asthma treatment in the ED [1].

Despite the research evidence, the uptake of MDI/spacers in pediatric emergency departments (PEDs) has been slow both in Canada and the United States [2]. A small number of international investigations that center on the successes in implementing MDI/spacers in PEDs [3,4] have demonstrated no changes in admission rates to the ward and intensive care unit (ICU), and have found that parent/child satisfaction is improved [5]. In addition, when asked, children and parents stated that they preferred using MDI/spacers to nebulisers.

In Canada there is a unique 'natural experiment' opportunity to study the adoption of MDI/spacer, because various PEDs across the country are at different stages of adopting this method of treatment. Focusing on the knowledge, attitudes, and practices of healthcare professionals regarding MDI/spacer use, Osmond and colleagues [6] conducted a survey in 10 PEDs across Canada and found that while most physicians and nurses believed that the evidence supported the use of MDI/spacers, few actually used this method of treatment in their personal practice. Hurley and colleagues [7] investigated the reasons for this discrepancy by comparing interview data with staff from a site that had adopted MDI/spacer use, to one that had not. With our study, we intend to further explore the issues raised in the study by Osmond and colleagues [6] (with more sites) and to explore the transferability of the findings from the Hurley study across early adopters, adopting sites, and sites yet to adopt MDI/spacers.

The overall objectives of this study were to determine the barriers and supports to implementing MDI/spacers into PED practice, and identify factors associated with early and late adoption of MDI/spacers in PEDs in Canada.

## Methods

### Design and sample

A comparative case study design [8] was used within the EDs of nine Canadian pediatric tertiary-care teaching hospitals. Case studies are appropriate when the boundaries between the phenomenon of interest and the context in which it occurs are not clear [8]. For this study, the phenomenon of interest was the adoption of MDI/spacers for the treatment of asthma, and while data collection occurred at the level of the individual practitioners, the unit of analysis was the individual PEDs. All of the hospitals belong to Pediatric Emergency Research Canada (PERC), a collaborative, nationwide pediatric emergency medicine research network. The EDs were classified into one of three categories based on stage of MDI/spacer implementation: 'early adopters,' 'adopting,' and those 'yet to adopt' [9]. Level of adoption was based on results from Osmond *et al.*'s study [6] in which members of ten Canadian PEDs were surveyed regarding MDI/spacer use, and was verified by a key informant. In the study by Osmond *et al.*, sites were categorized based on how individual emergency physicians at each site responded to a specific scenario given to them as part of the survey. For the purposes of the present study, 'early adopters' were those sites in which MDI/spacer had already been incorporated into routine practice for the treatment of mild to moderate asthma. 'Adopting' sites were those that were actively involved in switching from nebulised to MDI/spacer treatment, and 'yet to adopt' sites were those sites that exclusively used nebulised treatments and had not started a process to adopt MDI/spacers. We purposively sampled [10] from among the ten eligible PEDs so that there was equal representation from each of the three categories (the single site that was not chosen from the study by Osmond *et al. *had yet to adopt MDI/spacer use). Eligible participants included all PED physicians, ED registered nurses (RNs), respiratory therapists (RT) (in those EDs where RTs administered asthma treatment), as well as the medical and nursing directors in each of the departments.

### Ethical considerations and recruitment

The Health Research Ethics Board of the University of Alberta as well as the Ethics Review Boards of the nine participating institutions approved the study. An information letter outlining the details of the study, was sent to the PERC-affiliated research nurse or physician in each site, who in turn shared the information with all ED staff using methods of communication that were typical for their site (*e.g.*, email messages, posters, and memos). Informants self-selected to participate in the study.

### Data collection

Between March 2007 and March 2008, a Masters-prepared nurse (KO) with qualitative research experience and with no previous connection to any of the interviewees, collected data using both focus group interviews (n = 21) and individual interviews (n = 16). Interview participants were assured that their responses would remain confidential and anonymous, and that responses would be linked to 'categories' only, not individual settings. Two focus groups were conducted at each site: one with physicians, and one with RNs and RTs. Individual interviews were conducted with the medical and patient care directors at each site, either in person, or later by telephone. The semi-structured [9] interviews, lasting between 50 and 75 minutes, centred on the perceived barriers and facilitators to MDI/spacer use. Separate interview schedules [see Additional file 1] were developed for each level of adoption (early adopters, adopting, yet to adopt). Probing questions were used to help illuminate statements given by participants that were incomplete, vague, or ambiguous [11]. A court reporter, an individual trained in the verbatim recording of the spoken word, was used to record the focus group interviews, and both electronic and print copies of the transcribed interviews were produced [12]. A digital recording device was used to record the individual interviews, which were later transcribed. All interview transcripts were checked for accuracy and completeness using line-by-line comparison between the audio recordings and written transcripts. At each site, interviews were conducted until data saturation occurred, that is, until no new themes emerged from the data [10].

### Analysis

Two investigators (SS and KO) analyzed the data using a constant comparative [13,14] approach. Data collection and analysis proceeded concurrently. Data were managed using NUD*IST software (version N6, Qualitative Solutions and Research). Categories from the Ottawa Model of Research Use [15,16] (OMRU) were used to guide the development of the interview questions and topics covered, as well as the initial coding of the data and the organization of the emergent barriers and facilitators. Data were used to make cross-case (*i.e.*, pediatric ED to pediatric ED) and cross-category (*i.e.*, early adopter and yet to adopt) comparisons. Narratives relating to several major themes were developed.

Trustworthiness of our research data and analysis was guided by Guba and Lincoln's [17] criteria of credibility, confirmability, dependability, and transferability. We operationalized these criteria through a series of activities. First, we broadly sampled focus group participants to allow for multiple and diverse perspectives, as well as to ensure that we did not have an over-representation of data from particular professional groups (credibility criterion). Through the duration of the study, a comprehensive audit trail that documented all methodological decisions, conclusions, interpretations, and recommendations arising from the data was completed (confirmability). Furthermore, a complete inventory of all data collection and analysis products that includes written up detailed field notes, and theoretical and analytical memos that document developing thoughts about the data was also logged (dependability criterion). Finally, transferability was addressed by providing thick description of the EDs studied (while maintaining anonymity) with sufficient detail and precision to allow the reader to make judgements about applicability [18] to their respective settings.

## Results

There were nine tertiary level pediatric EDs comprising 150 participants. Three sites were already routinely using MDI/spacers in the ED ('early adopters'), two sites were in the adoption process, and the remaining four sites had not yet adopted the innovation. Category membership was based on results from the study by Osmond et al [6] and was verified by self report from a key informant. Table 1 outlines the number of participants by profession and category of MDI/spacer adoption, as well as the numbers of interviews in each category.

**Table 1:** Distribution of interview participants by profession and MDI/spacer adoption category

**Category**	**# of physicians**	**# of RNs**	**# of RTs**	**Total**	**# of focus group interviews**	**# of individual interviews**
**Early adopters Hospitals A, B and C**	23	27	4	54 (36%)	7	6

**Adopting Hospitals D and E**	15	16	2	33 (22%)	4	2*

**Late adopters Hospitals F, G, H, and J**	26	28	9	63 (42%)	10	8

**Totals**	64 (42.7%)	71 (47.3%)	15 (10%)	150	21	16

*at one 'adopting' site, the medical director and patient care manager elected to participate in the focus group interviews

Our findings are organized into thematic categories based on the OMRU [15] elements thought to influence research use: the evidence-based innovation (MDI spacers), the potential adopter, and the practice environment. A list of representative quotations from each thematic category has been provided [see Additional file 2].

### Characteristics of the evidence-based innovation

Through exploring the dissemination processes of the MDI/spacer, we acquired important information on several perceived features of the MDI/spacer that either hindered or supported its adoption by the participating PEDs including: cost, effectiveness, infection control, and impact on the patient. The site-specific barriers and facilitators identified by participants are summarized in Table 2.

**Table 2:** Barriers and supports to MDI/spacer use by site*

	**Early Adopters**			**Adopting**		**Yet to adopt**			
	
	**A**	**B**	**C**	**D**	**E**	**F**	**G**	**H**	**J**
**BARRIERS to MDI Use**									

Evidence-based Innovation									

Increased cost to the ED	x					x	x	x	x

Parental resistance	x	x	x	x	x	x	x	x	x

Extra time/extra work for nurses	x			x	x	x	x	x	x

Sterilization issues for the spacer devices				x			x	x	

Cost of the spacer to the patient		x				x			x

(Potential) Adopters									

Entrenched ideas/scepticism	x		x					x	

Not convinced by the research/no clear advantage						x	x	x	x

Practice Environment									

Language barrier (parents)						x	x	x	

Concerns about overtreatment at home by parents						x	x		

Institutional bureaucracy						x	x		

Lack of supplies or resources		x			x	x	x	x	x

Inconsistency of use in facility/region	x		x		x		x	x	

**SUPPORTS/FACILITATORS to MDI Use**									

Evidence-based Innovation									

Clear advantage acknowledged/'buy in'	x		x	x		x	x		

Perceived reduction in transmission of infection	x	x			x				

(Potential) Adopters									

Being involved in research			x		x		x		

Practice Environment									

Clear written protocol including MDI use				x			x	x	x

Encouraging staff participation in the change process	x			x					x

Having resources for patient education			x	x		x	x		x

Consistent treatment across department/facility/region	x				x	x	x		x

RT support		x	x		x		x		

Presence of a research champion	x			x	x	x			

Staff presented with rationale/evidence		x	x	x	x		x		x

Adequate resources/supplies	x			x		x	x	x	

Education for staff	x			x	x	x	x	x	x

*barriers and supports discussed by two or more sites

### Cost

The prevailing perception was that it was more expensive to deliver beta-2-agonists using a MDI/spacer than using a nebuliser. In fact, cost was the most significant factor perceived to shape the adoption process. Participants cited the extra time it would take to administer the medication and the cost of sterilization as factors contributing to the cost. While in several sites, sterilizing and reusing the spacers (up to five times) was seen as a way to reduce costs, in others, spacers were considered single use only and were either sold to patients or given away. Of note, participants in only one (early adopter) site thought that both treatment modalities took approximately the same amount of time, citing that the shorter time required for preparation and administration of the MDI/spacer made up for the time spent doing patient education. Regardless of who bears the cost of the intervention, all sites recognize the importance of resolving budgetary concerns prior to initiating adoption plans.

### Effectiveness

Despite being aware of the research evidence, many of the participants from the yet to adopt sites were sceptical that MDI/spacer was as effective as nebulisers. Most participants from early adopting sites and those sites engaged in the adoption process felt that using MDI/spacers were 'as good as if not better' than using nebulisation. Those who had already adopted MDI/spacers added that their personal experience validated findings from the research literature. Proper technique in administering the MDI/spacer was important to the participants in ensuring its effectiveness. Many participants in the 'early adopter' and 'adopting' sites felt that improper use of the MDI/spacer by patients contributed to the view that it was less effective in treating acute asthma than the nebuliser.

### Infection control

Regardless of their stage of adoption, most sites recognised that the use of the MDI/spacer was superior to the nebuliser in terms of infection control. The spread of disease via nebulisation was seen as a threat to patients and staff, but one that could be reduced significantly by the use of the MDI/spacer. The SARS (Severe Acute Respiratory Syndrome) outbreak that occurred in central Canada in 2003 was mentioned as a contributing factor to the adoption of MDI/spacer use in at least two sites.

### Impact on the patient

The MDI/spacer was generally seen to be a less intrusive treatment modality, and therefore less frightening to young children. Specifically, it is the mist, noise, and confining nature of the nebuliser treatment that make it unpleasant for this age group. Because MDI/spacer treatments take less time to administer (from the patients perspective), and parents are often asked to actively participate in the treatment, parents perceive it to be a less frightening treatment.

### Characteristics of the (potential) adopters

The clinicians from the sites participating in this study exhibited a range of knowledge, attitudes, and skills regarding the MDI/spacer. The majority of participants were aware of the results of research concerning the effectiveness of MDI/spacers, with physicians and respiratory therapists in general being more knowledgeable than nurses about specific studies. Even in adopting sites, there were individuals who had believed that nebulisation was more effective, especially for those patients who were in the moderate to severe range. The perception is that the majority of individuals, specifically physicians, needed to have 'bought-in' to the idea of using MDI/spacers for the adoption to take place. In order for this to occur, individuals needed to believe that the relative advantage gained by changing practice was 'worth' the energy and resources needed to make the change. Interestingly, buy-in on an individual level was often present at non-adopting sites, but because of other barriers and/or a lack of consensus, adoption had not occurred. Our findings on the characteristics of the adopters reflect the complexity of the MDI/spacer adoption process. While individual clinicians may be aware of the advantages of MDI/spacer use, the actually 'adoption' of MDI/spacers is actually an institutional or department decision because it requires support from different disciplines, as well as the allocation of different resources and products.

Participants expressed positive attitudes towards research in general regardless of their stage of adoption of MDI/spacers, yet some participants at early adopter and adopting sites were not entirely convinced by the literature supporting MDI/spacer use until they witnessed the outcomes for themselves. A number of participants at non-adopting sites expressed that research-based practice change that was done too quickly, and without adequate reflection, could lead to errors being made.

In some sites, MDI/spacer administration and/or patient education was performed by respiratory therapists, resulting in individual nurses being less comfortable performing these tasks. Respiratory therapists were considered a valuable resource in the five locations where they were permanent members of the ED team. Only two of the sites that either had adopted or were adopting MDI/spacer use did so without any RT support. Because of the high number of patients presenting with respiratory issues, having RTs assigned to these patients allowed nurses to focus their attention on other patients. Teaching and follow-up with patients were valued RT roles. At the sites where asthma treatment was not the sole responsibility of RTs, the nurses were more comfortable using MDI/spacers.

### Characteristics of the practice environment

While characteristics of the practice environment had the greatest potential to highlight existing differences between the nine sites, similarities among the sites were also found.

### Structural factors

The existence of a large number of part-time staff in the ED was mentioned as a potential barrier to maintaining consistent practices because it was difficult to communicate policy and practice changes with staff whose presence in the ED was infrequent. Many physicians who worked on a part-time basis were family physicians or pediatricians without specialty training in emergency medicine, and were perceived as being less up-to-date on the latest research in that area. The part-time nature of their positions within the ED made remaining current on departmental policies and procedures difficult. Often, they relied on advice or information from full-time staff to keep current. Interestingly, clinical practice variation was tolerated to different degrees among the nine sites.

High staff turnover in the PEDs resulted in difficulty in following up on practice change initiatives. Resources, such as more frequent staff inservices, may be needed to ensure that new staff are aware of practice changes. Staff shortages and the 'downloading' of tasks that were previously performed in inpatient units (due to bed shortages) contributed to a busy environment in the ED where there were many competing priorities. Noteworthy is that sites where the MDI/spacer was part of a written guideline, the practice was used more often and more consistently, with the exception of the one site where physicians were able to choose between nebulisers and MDI/spacers on the asthma guideline (regardless of severity). Interestingly, MDI/spacers in this site were never used.

Organizational bureaucracy exerted an influence in the majority of sites and was seen as a significant barrier (in terms of magnitude) to practice change. Participants expressed frustration at the amount of time and energy that was required to make modifications in clinical practice (policy changes). In order to conserve time, energy, and resources, staff recognized that only issues of the highest priority could be pursued and consequently, in some sites, it was recognized that adoption of MDI/spacers was not an urgent enough priority.

### Social and cultural factors

All sites characterized collegial relationships as cooperative and based on mutual respect. Furthermore, participants perceived relationships between different professional groups in the ED as more egalitarian than in other departments within the institution due to the way that work was organized in the ED. At all sites, nurses had the autonomy to assess patients and begin initial treatment.

Some sites, mainly early adopters, espoused a strong desire to be evidence-based and valued their reputation as a group that used 'cutting edge' practices. Other sites, while they acknowledge the value of being aware of the most current practice trends, were more 'cautious' in their adoption of new practices, preferring to see 'how things worked out' in other sites before attempting the practice change themselves. The willingness to 'take risks' was a characteristic of at least one of the early adopting sites.

Participants in adopter sites perceived that the presence of a professional (or professionals) within the department willing to champion the practice change was one of the most significant factors influencing in the adoption of the MDI/spacer. Most often, these individuals were respected clinicians by virtue of their experience and/or clinical expertise, had an interest in the specific clinical area (asthma), or had used MDI/spacers previously at another site. In two of the three early adopting sites, leadership at the clinical level was instrumental in achieving 'buy-in' by staff, and subsequently, adoption. Buy-in was achieved by providing both credible evidence and persuasive arguments for adoption of the practice. In sites that had not yet adopted MDI/spacer use, clinicians willing to champion the MDI/spacer research were recognized as being important to the change process, but either no one had committed to the role, someone had in the past but had not been successful, or someone was in the process of garnering support for the practice change, but had not yet achieved their goal. The consensus among participants was that a physician should be in the championing role, and it should be a person who has considerable credibility as a clinician.

### Patient-related factors

All of the sites expected parents to initially be resistant to the use of MDI/spacers in the ED. Participants from the early adopting sites acknowledged that parents were sceptical about the use of a treatment they had already used at home and had presumably 'not worked'. In particular, parents and children who were frequent ED visitors were perceived to be the most fervent in their resistance. Parents also associated the use of the nebuliser with the administration of oxygen, and consequently doubted that any treatment without oxygen would be helpful. Participants in early adopter sites said that while it took extra time to educate and reassure parents of the effectiveness of the new treatment, most were eventually convinced when they understood why it was being used, and witnessed the outcomes for themselves.

In sites where adoption had not taken place, the resistance expected to come from parents was looked at more negatively and more challenging to overcome. Nurses saw themselves as having to 'take the brunt' of the complaints by parents, predicting that they would be put in the difficult position advocating to back the 'old' delivery system on behalf of the family. Despite the broadly expressed concern that parents would 'over treat' their children at home having seen a greater number of puffs being administered in the ED, there was no evidence, experiential or otherwise, to support that concern, and some participants noted that actually the opposite situation occurred occasionally. Early adopter/adopting sites perceived an increase in patient empowerment and confidence, and a decrease in parental anxiety after having been shown how to manage their child's asthma effectively with the MDI/spacer.

The severity of a patient's asthma had a bearing on which method of treatment was used with 'mild' and 'moderate' patients receiving medication via MDI/spacer, and 'severe' patients receiving nebulisation. Of note, in some sites, physicians believed that there was a tendency towards overestimating the severity of the patient's condition at triage, resulting in a higher than necessary use of nebulisation.

Regardless of their stage in the adoption process (early adopter, adopter or non-adopters/yet to adopt), many sites identified the same barriers and supports to MDI/spacer adoption. The difference between early and late adopting sites was that early adopter sites dedicated resources to overcome adoption barriers. In order for resources to be directed towards the goal of adoption, consensus had to be reached among a majority of the medical staff. Most often, this process was lead by one or more individuals who championed the cause within the department, presenting the research evidence to the other staff members. Both a lack of consensus among staff and the lack of a research champion were the biggest barriers to adoption in late adopting sites. In all late adopting sites, there were significant numbers of staff, both physicians and nurses, who saw no clear advantage to adopting MDI/spacers.

Parental resistance was broadly cited as a potential barrier to adoption, however, in early adopting sites, steps were taken to ensure that this barrier was adequately addressed. Staff attended organized educational sessions, educational materials were prepared for patients and their families, and in some cases, a campaign was launched to notify the broader community of the practice change. Staff recognized that in order to convince parents of the effectiveness of the new practice, they themselves had to be confident in the practice change.

The perception that administering medication via MDI/spacers took more time and effort was prevalent among all three groups. In early adopting sites, nurses were willing to invest time to educate patients about MDI/spacer use as it would 'pay off' later with better asthma control and self-administration of medication in the ED. Staff at one early adopter site were convinced that MDI/spacer administration actually took less time than nebulisation.

The final major barrier to MDI/spacer adoption was cost. Because nebulisation would still be used for the more severe cases, some argued that 'doubling up' on equipment would be wasteful. Also, some participants believed that there were other priorities within the department that were more deserving of a portion of the limited available resources.

Sites that had successfully adopted the MDI/spacers had some common strategies such as participation of all professional groups and having a well-planned and executed educational component. Furthermore, having continuity of practice within the facility (*i.e.*, same protocol in ED and in-patient units), between facilities or between the facility and community physicians made the process easier because patients received consistent information that was reinforced. As well, including MDI/spacer use as part of a guideline or protocol also facilitated successful adoption in these sites. Essentially, the sites that had successful adopted the MDI/spacer were able to recognize the unique characteristics of the innovation, adopters, and practice environment at their particular ED and successfully use these attributes to facilitate adoption.

## Discussion

The findings from our case study suggest how barriers and facilitators interact with each other in complex ways (Table 2) to produce different outcomes in each of the study EDs. In fact, there was not a clear pattern with respect to the barriers and facilitators in terms of stage of MDI/spacer adoption by EDs. Noteworthy is that the early adopter sites reported having many of the same barriers that sites that had yet to adopt the innovation had, however, early adopter sites were motivated to find ways to overcome these barriers. The identified barriers and facilitators related to: attributes of the innovation (MDI/spacer), such as perceived ease of use, clear advantages of MDI/spacers and cost; attributes of the practice environment including both structural (*e.g.*, staffing issues, organizational bureaucracy) and social factors (*e.g.*, presence of a research champion, autonomy); and attributes of the individual clinicians working within the EDs including elements such as entrenched ideas and scepticism. This complex array of factors at multiple levels shaping the adoption process mirrors the findings of Denis and colleagues [19]. They suggested that adoption processes followed different paths and factors that facilitated adoption at one site may hinder adoption in another site given the complex interplay of individual, contextual, and historical factors.

Furthermore, our findings suggest that the adoption of MDI/spacers is not easily reduced to the decision by an individual ED physician. Rather, the decision to adopt MDI spacers requires support and decisions at levels ranging from the individual practitioner, parents, department, institution, and regional levels. Our findings illustrate that individual clinicians cannot simply decide to change their clinical practice decision making and prescribe the use of MDI/spacers. Our findings offer that this is an ED decision and the 'adopter' *per se *is the ED, not the individual clinician. The decision is not straightforward and does not involve exclusively practitioners becoming convinced only of the strength and rigour of the scientific evidence promoting the efficacy of this innovation, but rather, the organizational decision to adopt MDI/spacers is a complex decision that requires savvy and persuasion at multiple levels (clinician, department decision-makers) and the allocation of significant resources (human, equipment, and financial) in order to facilitate success. The complexity of this organizational decision, by default, suggests that planned change strategies are required to facilitate the adoption of MDI/spacers. Our findings suggest that passive techniques where there are no champions in place and the strength of the research evidence is the sole motivator for adoption are not effective. In essence, knowledge is not enough to facilitate adoption. In particular, clarity over who is going to bear the cost of the MDI/spacer and harnessing the support of leadership and a champion were critical forces in garnering adoption in our study. Although the decision to 'adopt' MDI/spacers is a multi-level institutional or department decision, foundational to successful adoption is individual clinician trust in the research that outlines the benefits of MDI/spacer use.

The findings from this study complement the work of Osmond and colleagues [6] who surveyed Canadian multidisciplinary ED healthcare professionals regarding their practices, beliefs and barrier to metered-dose inhaler/spacer use. Osmond's findings highlighted that professionals from sites that used and did not use the MDI/spacer had positive beliefs and knowledge about the innovation. Furthermore, physicians from both adopting and non-adopting sites expected equal or enhanced clinical outcomes with MDI/spacer use; however 70% of physicians did not use MDI/spacers for treatment of pediatric asthma exacerbations. Osmond's work highlights the fact that awareness of the benefits of MDI/spacers is not enough to shape adoption process. The findings of our study provide rich detail about the complex array of individual, innovation and practice environment elements that influence this practice change.

In addition to complementing Osmond's work, our findings also build upon the work of Hurley and colleagues [7]. They explored Canadian ED healthcare professionals' perceptions associated with the use or non-use of MDI/spacers for the delivery of beta-agonist respiratory medications in two teaching hospitals. They discovered the main impediments to be increased workload, increased equipment costs, myths about the superiority of nebulisation, and interprofessional conflict. While there are parallels in terms of some of the identified barriers to MDI/spacer adoption, our findings revealed a more dynamic picture with early adopter and yet to adopt sites sharing some of the barriers, yet early adopter sites created/generated support and leadership to overcome the barriers. Through a more robust research design and a larger sample size, we were able to build upon Hurley's findings and learn that clarity about cost implications and the support from leadership and a MDI/spacer champion can be integral components to successful adoption.

### Study limitations

While this study sheds light on the factors that shaped the adoption process of the MDI/spacer, the results must be interpreted cautiously because individual responses were used to developed thick descriptions of a unit-level phenomenon (adoption of the MDI/spacer). Although focus group interviews are an effective technique to acquire data on unit-level phenomenon, and much effort was exerted to ensure a broader representation in the composition of the focus group participants, readers must be mindful that individual perspectives were acquired. While we were able to identify the barriers and supports to implementation of the MDI/spacers, we collected data on several important contextual factors, such as ED census, and decision-making structure; however, in order to protect anonymity, specific data on these elements cannot be shared at the individual ED level. We did not interview parents to explore their perspective of MDI/spacer use. While they could have been considered adopters of the innovation because they must give consent for medical intervention, we limited our focus to how they influenced clinician behaviour. The final limitation of our study is a temporal one. It must be noted that we were not intervening and studying the effects of various strategies to facilitate MDI/spacer adoption, rather, we were retrospectively exploring the adoption processes for EDs that had either already adopted or were adopting the MDI/spacer, or exploring potential factors for sites that had not yet adopted the MDI/spacers. Thus, limitations arise when asking participants to reflect upon events that happened, in some cases, several years ago. For example, participants in sites where adoption had taken place may have unintentionally justified their present practice by 'glossing over' the main barriers they had experienced in the past. Having already adopted the practice, adopters could have been 'convincing themselves' that the path chosen was the correct one. However, through the use of focus groups, we believe that this limitation is minimized through the garnering of multiple perspectives on the process.

## Conclusion

The dissemination of research evidence rarely is sufficient by itself to improve healthcare and specific interventions are needed to address local barriers and facilitators [20]. This is true with MDI/spacer adoption because, despite strong research evidence that MDI/spacer can produce results equivalent to nebulisers, adoption of MDI/spacers for the treatment of mild and moderate asthma has been slow. Building upon the findings of key articles in the field [16,17], we studied the natural evolution of diffusion patterns of MDI/spacers for the treatment of mild to moderate asthma in children in nine PEDs. Through this research, we developed new knowledge about the social and political nature (*i.e.*, in terms of resource allocation and presence of champions) of the adoption process as well as complexity in terms of the number of factors and levels that innovation adoption demands. Clinical treatment and management of children's asthma exacerbations are engrained decisions and behaviours that are shaped by factors at the individual practitioner, department, and institutional levels. Adding to this complexity, parental perceptions and expectations weighed heavily into healthcare professionals' decision making processes and the overall adoption process. Generally speaking, the findings make important contributions to the complexity of innovation adoption processes on two fronts. First, awareness or knowledge of the innovation is not enough to change practitioner behaviour. Rather, of note, most practitioners knew of the merit of MDI/spacer use, however, this knowledge was not enough to independently propel successful adoption because of the magnitude of other unit level barriers. Second, there were no 'magic bullets' or patterns of barriers and/or facilitators that consistently led to adoption success at each of the adopter/adopting sites. Rather, the unit/departmental barriers and facilitators interacted and interplayed with historical, contextual, and cultural values at each site. Our findings represent important knowledge for EDs preparing to implement MDI/spacers for asthma treatment, and potentially other innovation adoption decisions that are based upon strong research evidence. This study demonstrates the value of assessing the unit-specific barriers and facilitators prior to planned implementation change initiatives in order to tailor implementation strategies.

## Competing interests

The authors declare that they have no competing interests.

## Authors' contributions

SDS provided leadership and coordination in the design and conduct of the study, participated in data analysis and interpretation, drafted and edited the final manuscript, and approved the final submitted manuscript. MHO conceived the study, contributed to the overall study design, participated in data analysis, assisted in drafting and editing the manuscript. KAOL carried out data collection, participated in analysis, and helped to draft and edit the manuscript. IDG, JG, and TK contributed to study conception, and participated in critically appraising and revising the intellectual content of the manuscript. All authors read and approved the final manuscript.

## Supplementary Material

Additional file 1 **Interview Schedules**. Separate interview schedules for focus group interviews with staff from 'Early Adopting', 'Adopting', and 'Yet to Adopt' sites. Click here for file

Additional file 2 **Representative Quotations**. A list of verbatim quotations illustrating each of the emergent themes. Click here for file
