# Supplementary material for: Barriers and supports to implementation of MDI/spacer use in nine Canadian pediatric emergency departments: a qualitative study
Source: Implement Sci. 2009 Oct 13;4:65. doi: 10.1186/1748-5908-4-65 (PMC2766417; doi:10.1186/1748-5908-4-65)
Supplement: Additional file 1 — Interview Schedules. Separate interview schedules for focus group interviews with staff from 'Early Adopting', 'Adopting', and 'Yet to Adopt' sites. [file 1748-5908-4-65-S1.DOC]

Study Title: Barriers and supports to implementation of MDI/spacer use in nine Canadian pediatric emergency departments: a qualitative study

**INTERVIEW SCHEDULES**

Interview questions for ‘Early Adopting’ sites:

We are very interested in learning about your institution’s experience with MDI/spacers.

1.0 How are children who present with an asthma exacerbation normally managed/treated in your institution’s ED?

2.0 Tell me about the work environment of the emergency department—**in relation to** willingness to change practice.

Probe—what would you tell a new staff member about how ’things are’ in the ED?

Probe—what are the ‘unwritten rules’ that relate to introducing new procedures?

1. What do you **know** about MDI/spacers and acute asthma treatment in the ED?
   1. Where do you get this knowledge/information from (*e.g.*, Cochrane, colleagues, published literature)?
   2. What are your perceptions about your colleagues’ knowledge regarding the evidence on MDI/spacers?
   3. How effective do you think MDI/spacers are?
2. What do you **think** about MDI/spacers and acute asthma treatment in the ED?

4.1 How about your colleagues?

4.2 What about other professional groups within the ED?

5.0 Tell me about how the shift to MDI/spacers happened in your ED.

5.1 What was the response when the MDI/spacers were introduced in your ED?

5.2 Were there any people particularly involved in the adoption of the EDs? What did they do?

5.3 How were they introduced in your setting? Did you get to try the spacers before they were formally adopted?

5.4 Based on the results of initial attempts were changes subsequently made to how MDI/spacers were initially introduced at your ED?

6.0 What is your assessment about how well your emergency department has adopted the use of the MDI spacer for treatment of asthma exacerbations?

6.1 How long did it take to get people to accept/use the MDI/spacers?

6.2 What were the challenges and barriers that made adoption difficult?

Probes for barriers: i) sterilizing/reusing; ii) cost to the ED; iii) parental expectations to use the nebulizer; iv) absence of a physician champion; v) physicians who do not believe that MDI/Spacers are as effective as nebulizers; vi) concerns of increasing nurse workload; vii) ED physician inertia to change; viii) ED nurses inertia to change.

6.3 What facilitated the adoption of the MDI/spacers?

7.0 What infrastructure support was necessary to facilitate the adoption of the spacers?

Probes *e.g.*, Education sessions? Reminder systems? Champion/opinion leaders? Audit and feedback? Posters? Other?—please explain

8.0 What changes have there been as a result of adoption of MDI/spacers?

Probes:

8.1 Changes in physician practices?

8.2 Changes in nursing practices?

8.3 Changes in policies or procedures?

8.4 How have parents responded to the change? What has been their reaction?

8.5 What about the financial aspects of this change?

8.6 What has been the response from other disciplines? What? Which disciplines? (*e.g.*, pharmacy)

8.7 What about unintended results or unintended spin-offs? What?

9.0 Your ED adopted MDI/spacers when other EDs across the country have not—what are your thoughts on the reasons for this?

Thank you for your thoughtful answers to my questions. Are there **any other comments** you would like to make that might help us understand the use of MDI/spacers in your institution?

May we approach you again should we have any questions about what you have told us?

Interview questions for ‘Adopting’ sites:

We are very interested in learning about your institution’s experience with MDI/spacers.

1.0 How are children who present with an asthma exacerbation normally managed/treated in your institution’s ED?

2.0 Tell me about the work environment of the emergency department—**in relation to** willingness to change practice.

Probe—what would you tell a new staff member about how ’things are’ in the ED?

Probe—what are the ‘unwritten rules’ that relate to introducing new procedures?

3.0 What do you **know** about MDI/spacers and acute asthma treatment in the ED?

3.1 Where do you get this knowledge/information from (*e.g.*, Cochrane, colleagues, published literature)?

3.2 What are your perceptions about your colleagues’ knowledge regarding the evidence on MDI/spacers?

3.3 How effective do you think MDI/spacers are?

4.0 What do you **think** about MDI/spacers and acute asthma treatment in the ED?

4.1 How about your colleagues?

4.2 What about other professional groups within the ED?

5.0 Tell me about how the shift to MDI/spacers is occurring in your ED.

5.1 What has been the response to the introduction of MDI/spacers in your ED?

5.2 Are there any people particularly involved in the adoption of the EDs? What are they doing?

5.3 How are MDI/Spacers being introduced in your setting? Do you get to try the spacers before they are formally adopted?

5.4 Based on the results of the **earliest** attempts, have changes been made to how MDI/spacers are being introduced at your ED?

6.0 What is your assessment about how well your emergency department is adopting the use of the MDI spacer for treatment of asthma exacerbations?

6.1 How long is it taking to get people to accept/use the MDI/spacers?

6.2 What are the challenges and barriers that made adoption difficult?

Probes for barriers: i) sterilizing/reusing; ii) cost to the ED; iii) parental expectations to use the nebulizer; iv) absence of a physician champion; v) physicians who do not believe that MDI/Spacers are as effective as nebulizers; vi) concerns of increasing nurse workload; vii) ED physician inertia to change; viii) ED nurses inertia to change.

6.3 What facilitates the adoption of the MDI/spacers?

7.0 What infrastructure support (in your opinion) is necessary to facilitate the adoption of the spacers?

Probes *e.g.*, Education sessions? Reminder systems? Champion/opinion leaders? Audit and feedback? Posters? Other?—please explain

8.0 What changes are being made as a result of adoption of MDI/spacers?

Probes:

8.1 Changes in physician practices?

8.2 Changes in nursing practices?

8.3 Changes in policies or procedures?

8.4 How have parents responded so far to the change? What has been their reaction?

8.5 What about the financial aspects of this change?

8.6 What has been the response from other disciplines? What? Which disciplines? (*e.g.*, pharmacy)

8.7 What about unintended results or unintended spin-offs? What?

9.0 Your ED is adopting MDI/spacers when other EDs across the country are/have not—what are your thoughts on the reasons for this?

Thank you for your thoughtful answers to my questions. Are there **any other comments** you would like to make that might help us understand the use of MDI/spacers in your institution?

May we approach you again should we have any questions about what you have told us?

Interview questions for ‘Yet to Adopt’ sites:

We are very interested in learning about your institution’s experience with MDI/spacers.

1.0 How are children who present with an asthma exacerbation treated normally at your institution’s ED?

2.0 Tell me about the work environment of the emergency department—**in relation to** willingness to change practice

Probe—what would you tell a new staff member about how ’things are’ in the ED?

Probe—what are the ‘unwritten rules’ that relate to introducing new procedure?

3.0 What do you **know** about MDI/spacers and acute asthma treatment in the ED?

3.1 Where do you get this knowledge/information from (*e.g.*, Cochrane, colleagues, published literature).

3.2 What are your perceptions about your colleagues’ knowledge regarding the evidence on MDI/spacers?

4.0 What do you **think** about MDI/spacers and acute asthma treatment in the ED?

4.1 What about your colleagues?

4.2 What about the other professional groups within the ED?

1. There are some EDs in Canada that have adopted MDI/spacers for the treatment of acute asthma exacerbations—are you thinking about adopting it?
   1. If not, what are the issues around that?
   2. What would be the barriers/facilitators (regardless of whether or not you are thinking of adopting it)?

Probes for barriers: i) sterilizing/reusing; ii) cost to the ED; iii) parental expectations to use the nebulizer; iv) absence of a physician champion; v) physicians who do not believe that MDI/Spacers are as effective as nebulizers; vi) concerns of increasing nurse workload; vii) ED physician inertia to change; viii) ED nurses inertia to change.

6.0 From your perspective, if an administrative decision was made to adopt MDI/spacers as the ‘treatment of choice’ for children with asthma exacerbations, in your institution what would need to happen to make the change success? (*e.g.*, reminder systems, opinion leaders, audit and feedback, etc.)

Thank you for your thoughtful answers to my questions. Are there **any other comments** you would like to make that might help us understand the non-use of MDI/spacers in your institution?

May we approach you again should we have any questions about what you have told us?
