# Supplementary material for: Barriers and supports to implementation of MDI/spacer use in nine Canadian pediatric emergency departments: a qualitative study
Source: Implement Sci. 2009 Oct 13;4:65. doi: 10.1186/1748-5908-4-65 (PMC2766417; doi:10.1186/1748-5908-4-65)
Supplement: Additional file 2 — Representative Quotations. A list of verbatim quotations illustrating each of the emergent themes. [file 1748-5908-4-65-S2.DOC]

Study Title: Barriers and supports to implementation of MDI/spacer use in nine Canadian pediatric emergency departments: a qualitative study

**Representative quotations**

| **OMRU Element: Evidence-based Innovation** | |
| --- | --- |
| **Influencing Factor** | **Quotation** |
| Cost of the spacers | ‘In my experience the barriers to this adoption has not been the physicians, it has been the administration and the other departments of the hospital. It has been a cost issue**.’ Y/PH**  ‘When we wanted to switch primarily to MDI and Spacer, the issue came up that there would be a significant cost associated with the Spacer devices. Also, supply was an issue: How can we actually roll this out when we don't have a supplier that can give us the 20,000 spacers we are going to go through in the next year any time soon.’ **E/PH** |
| Extra workload | ‘I think a big one [barrier] actually would be nursing time because right now they slap on the nebuliser and basically the child screams—and the nurses can go off and do other things, whereas this at least I think may require the nurse to go back into the room for teaching or something or even to stand there while they are doing it, although having said that, it probably takes one fraction of the time of a nebuliser.’ **Y/PH**  ‘The workload, even if it's a false perception that the workload is increased, good luck to you. That's the key. People have to feel like their workload improved with this change**.’ Y/PH** |
| Sterilization | ‘One big issue that came up surrounded the fact that we were previously sterilizing them and using them multiple times, and then it came down that we couldn't do that any longer, that they are single patient use, and then all of a sudden there was this huge cost associated with it. So that was a bit of a barrier at the beginning.’ **E/PH** |
| Cost to the patient | ‘We have a lot of poor families who won't buy them, we have a lot of irresponsible families who will lose it the minute they walk out the door and be coming back and wanting another one from us. And if they have to buy it when they're in the community, then people aren't going to buy it; they'll just come back here and get one from us.’ **A/RN**  ‘You do not have any way to factor in abuse of the system, like, when people bring three or four kids and everybody is getting an MDI and going home with $200 worth of products? Like, you would have to have some type of guidelines then.’ **Y/RN** |
| Conflicting/ unconvincing evidence | ‘I mean not being an expert in this realm but it sounds to me that there's a lot of data missing prior to really making a change for a treatment that is equally effective, is my understanding, but there's a lot of other data that, you know, might be why there’s been a lot of reluctance**...’ Y/PH**  ‘If you are being asked to change practice based on evidence that's equivocal, that's going to be hard to do**.’ Y/PH**  ‘I think there's the problem that lay with the spacers and the masks. It's equivalent but doesn't have a huge definite advantage.’ **Y/PH** |
| Issues around technique | And it's quicker for the parents as long as they're shown how to use it properly. But I think it's only as effective as the person using it. And that's the key issue**. Y/RN**  I think that the real push was because of the concerns about proper use in the community and just a great opportunity to teach the parents and children how to use these devices properly to get the best bang for their buck, so my understanding was that it was the teaching opportunity and the lack of—and the equivalent sort of therapeutic efficacy which led to adopting them. **A/PH** |
| Comparisons to the nebuliser | ‘Some of us feel a little bit more like we're doing something for the parents that they couldn't have done, but you know with an MDI, the implication is going to be if you'd used this differently at home you might not have had to come in.’ **Y/PH** |
| Infection control | ‘Probably the biggest thing for me is the notion that it doesn’t nebulise influenza virus around the room. That’s probably the biggest most convincing piece of evidence I’ve heard recently.’ **A/PH**  ‘Nobody's even talked about what it means to be standing there when you smack a nebuliser on some kid that you know, when it's like all their RSVs, the other virus, right. You're right there; the parent's right there.’ **Y/PH** |
| Severe Acute Respiratory Syndrome (SARS) | ‘I think we were already well along the way in terms of changing practice before SARS hit. I think basically SARS just sort of mandated it was 100 percent rule at that point in time. But I would say that we were probably 60 to 75 percent along the way to change before it even hit.’ **E/PH**  ‘You know, I worry about SARS, but not as much as I worry about getting something else. Like to me, TB, like a really bad meningitis. I don't want to have to take prophylactic antibiotics because I was exposed to...a new virus, but SARS, it's too bad that more people die every year of TB, pneumonia, those sorts of things and those things concern me more. ‘ **Y/PH** |
| Convenience | ‘My little quiet survey at Triage tells me that they don't take them [spacers] out. They use them at home, but never outside, because it makes them look like they are geeks, or whatever.’ **E/RN**  ‘They're bulky to carry around and lose convenience that way.’ **E/PH** |
| Complexity | ‘I think that's just because everyone is familiar with it anyway, so I guess it wouldn't be as big an issue as it would be for somebody who is using something totally different.’ **Y/PH** |
| Patient impact | ‘I think [the MDI and spacer] works just as good if not better, and you definitely don't have as much anxiety from the child for the most part.’ **A/RN**  Think of it yourself. If you are having trouble breathing and somebody comes at you with a foul smelling misty thing that makes noise and shoves it over your face, it would be terrifying.’ **E/RN** |

**E** = Early adopter; **A** = Adopting; **Y** = Yet to adopt; **RN** = nurse/respiratory therapist focus group/interview; **PH** = physician focus group/interview

**Representative quotations (con’t)**

| **OMRU Element: Potential Innovators** | |
| --- | --- |
| **Influencing Factor** | **Quotation** |
| Knowledge of the literature | ‘I think most of us are familiar—most of us—in the full-time group anyway, familiar with the literature, are aware of its equal efficacy to nebulisers and we have been waiting for this to happen.’ **Y/PH**  ‘He [the RT] provided us with articles saying that this is what you should be doing. And at that point I know it was conclusive, so he had research then. And—but our physicians weren't convinced, wanted to go with what we currently use.’ **Y/PH** |
| Attitude towards MDI/spacer | ‘I would have to say 99 percent of our physicians are pro-spacer. They're all for it because evidence has proven that there's no difference in efficiency of administration.’ **Y/PH**  ‘To be honest, when the idea [use of MDI/spacers] was first introduced, people were not too pleased.’ **E/PH** |
| Attitudes towards research | ‘And so it's a bit of a balancing act between that and a lot of the younger crew that are coming up who are very well attuned to the current literature. And it's just sort of a balance. So I think we’re all fairly open-minded. Sometimes things will change faster than others; sometimes it's slower.’ **Y/PH**  ‘The doctors read the research. They know that it's the right thing to do, or that it’s been proven that it's effective, so they'll be behind it for that reason.’ **Y/PH** |
| Attitudes towards change | ‘I think in general in medicine we're slow to change. And we kind of get locked into our practice patterns and people do tend to stick with them.’ **Y/PH**  ‘We’re not followers, here. We’re innovators.’ **E/RN**  ‘I’d say we’re probably… we’ve had to change… the emergency department is usually the first place where clinical practice starts to get changed. We’re very much involved in research down here.’ **Y/PH** |
| Experiential evidence | ‘We are given [research evidence] and it still doesn't have the same effect as being aware of my own outcomes.’ **E/PH**  I think that was probably more important than knowing the literature was having seen it work in practice.’ **E/PH** |
| Proficiency | ‘I'm actually more comfortable doing a wet treatment just because that was my experience and really I haven't had any formal education on using the puffer.’ **E/RN** |

**E** = Early adopter; **A** = Adopting; **Y** = Yet to adopt; **RN** = nurse/respiratory therapist focus group/interview; **PH** = physician focus group/interview

**Representative quotations (con’t)**

| **OMRU Element: Practice Environment—Structural Factors** | |
| --- | --- |
| **Influencing Factor** | **Quotation** |
| Staffing issues | ‘And the other main factor is we do have quite a variety of types of physicians working in our particular emergency department and that may complicate things in terms of information transfer. Some of us are aware through the emails that we read all the time of the changes in education whereas some people are here once or twice in a ten week block and that population might be more difficult to access.’ **Y/PH**  ‘It’s reasonable to think that the people who work less hours will maybe take a little longer to get into the groove of things just because you’re not present as much as people who are here more often.’ **A/RN**  ‘What I think is really important too is continuing education because you might train and do inservices on all these RTs and nurses but the turnover rate is so high, especially in Emerg, that when any group starts, there is never a follow-up or orientation for that group so it kind of needs to be consistent all the way across.’ **Y/RN** |
| The use of RTs | ‘We’re a fairly busy department, so we usually try and take advantage of the RTs if we can because they are the ones that give the ongoing care afterwards. Sometimes when we have a patient come in and we know that they need Ventolin and we don’t feel that there’s time to wait, we will give the first dose and then get the RTs to continue care.’ **E/RN**  ‘I worked in a hospital that had RTs to do everything respiratory-wise and everything, and it was fantastic, so bring it on.’ **Y/RN** |
| Organizational bureaucracy | ‘The hospital itself I think it's very difficult to change anything at a hospital level. You have to go through seventeen different committees and sell your soul to several devils for several years to implement any change to an order sheet. If you want to add something to an order sheet, I don't know who you need to go talk to, but it is not going to happen in a week or two, it might be a year or two.’ **Y/PH**  ‘The bigger the system, the more complicated the transition. So if you’re in a smaller community hospital it’s way easier to make stuff happen, I think.’ **A/PH** |
| Protocol/  guide-line availability | ‘It’s [asthma protocol] not well defined. That’s another problem. There’s a little bit of a discrepancy right now. And what we are trying to clean up at the same time is to really come up with a clear asthma protocol which includes the MDI and which includes the use of the medical directive for MDI in triage.’ **Y/RN**  ‘I would definitely see moving it right into the care map. You know, the care map’s the driver right now, and if it becomes part of the care map just like—you know, I guess I really believe that if you just make it practice, and if it’s written down as a rule, and if you put it in the care map it becomes a rule, because you have to obey—you have to work by the care map.’ **Y/RN** |
| Limited resources | ‘And it’s all because—just to clarify, because we don’t have the resources to audit this stuff, and go back and really do that sustained implementation that perhaps we would love.’ **Y/RN**  ‘This place is super busy! You have to take into account the context of where it is being done. You have a thousand other things going on. In some ways, the asthmatics who are moderate are on autopilot... That is an easy patient to have, in a lot of ways, because they are simply being looked after. That is not somebody that you have to be in the room a lot with. That moderate group can easily languish while awaiting reassessment. It is easy to see how that happens when you have twenty other patients that you are dealing with.**’ E/PH** |
| Higher acuity | ‘Emergency was the ABCs, the airway breathing circulation stabilized to the OR, to the floor. Now it’s gone way beyond that and you know we’re instituting certain things that would have normally been done in the intensive care unit setting because there’s inability to get the patient into those beds.’ **Y/RN** |

**E** = Early adopter; **A** = Adopting; **Y** = Yet to adopt; **RN** = nurse/respiratory therapist focus group/interview; **PH** = physician focus group/interview

**Representative quotations (con’t)**

| **OMRU Element: Practice Environment—Social Factors** | | |
| --- | --- | --- |
| **Influencing Factor** | | **Quotation** |
| Participation in research | | ‘So [if] you do research or a paper, you have a lot of local initiative and personal investment in it... once you start doing research on it, that makes it much easier to incorporate.’ **Y/PH**  ‘[I] think sometimes when you introduce a study it just becomes part of the practice. And so with those hospitals that have been fortunate enough to have that can transition a little bit easier**.’ A/PH** |
| Collaboration | | ‘It’s a very different environment where people are working alongside each other all the time so there has to be that mutual respect. You can’t—this is more complex in terms of change.’ **A/PH**  ‘And just—I think it’s just—that’s the main reason why it happened. And also, we also have a group that’s very—pretty progressive and open, open minded and not stuck in their old ways kind of thing. I think that’s the personality of the group working here. I just think that that just made it even easier.’ **E/PH** |
| Autonomy | | ‘I think also for the physicians it's a very different structure. They are contractually employed by the hospital. They have a certain comfort level with their independence as a practitioner. We're independent practitioners but we are employees of the hospital. So if the hospital says you are now going to do electronic patient ordering unless you don't...unless you do it you...well you need to leave. The physicians have a little bit more...they have a little more wiggle room.’ **E/RN**  ‘So, it is basically clinical judgment. The nurse at the front decides whether or not she is going to give nebulisation and the three masks in a row with Atrovent, or whether the child gets MDI. That decision is made before the physician has seen the patient, in the vast majority of cases.’ **E/PH** |
| Clinical variation | | ‘It [clinical variation] was viewed as a positive thing in the past, but I think people are understanding more that we need guidelines and for people to follow guidelines on a regular basis, so we are in the clinical practice guidelines era, and people feel that it’s needed.’ **Y/PH**  ‘The older [the physicians] are, the less receptive they are to the protocol. The younger ones have it done more readily. The others prefer to see their patients themselves.’ **E/PH**  ‘Here, there’s a lot of variation in practice. And unfortunately, the physicians are very hesitant to commit to like a clinical practice guideline. They will like—they may or may not follow the same basic approach to, say, asthma, but they all have their little [idiosyncrasies]...’ **Y/RN** |
| Best practice | | ‘And it's nice to know that you're treating some people with the latest evidence and best practice methods. That's very comforting to know.’ **A/RN**  ‘But I think we’re a lot more flexible now. I think we’re moving towards a greater emphasis on evidence and a less emphasis on tradition.’ **A/PH** |
| Competing priorities | | ‘Certainly one of the realities in our division is that we have many different things going on all the time and so in terms of people who are going to put time and energy into making the change or advocating for it, we don't really have, you know, somebody who seems to have gone to the wall to champion the change. Not that people aren't interested, they are busy doing many other things.’ **Y/PH**  ‘Whatever benefit there is, if it was an equal benefit for bronchiolitis and an equal benefit for asthma, and we only had the resources, whether they be financial, human or otherwise to take one or the other, we’d take the bronchiolitis.’ **Y/PH** |
| Research champion | ‘Unless you have someone to actually head it up and take the role and head it, it will never go anywhere. And you need some—you need a doctor to do that. It would never have come from a nursing or RT level. It needs to come from a doctor level.’ **E/RN**  ‘Change in this organization, like many others, it takes time, and it takes a champion to move it forward. And when I say time it’s only because of the culture, and also because of the need administratively both through different committees, and people that are involved in those changes both within and outside the division.**’ Y/PH**  ‘For something like MDIs, if there isn't somebody to champion it, it's almost this knowledge transfer wall. We all know it, but to actually make it happen, it's a whole other science. If you don't have someone pushing it through...’ **Y/PH** | |
| Pediatric expertise | ‘I bet they [other hospitals within the city] see fewer true moderately severe asthmas. Because I think if your kid is really sick in the city, you try and come here first. But, I mean, I know they see them because we get them from them. So it is not like there is zero, but I am sure that is part of it. But it seems to me when we see adult trainees come in and they have a very different approach to asthma than we do.’ **Y/PH**  ‘We still always have the advantage that we are considered the gold standard for care. So if someone else does something and then they get sent here, they usually get sent here because it's not working.’ **Y/PH** | |

**E** = Early adopter; **A** = adopting; **Y** = Yet to adopt; **RN** = nurse/respiratory therapist focus group/interview; **PH** = physician focus group/interview

**Representative quotations (con’t)**

| **OMRU Element: Practice Environment—Patient-related Factors** | |
| --- | --- |
| **Influencing Factor** | **Quotation** |
| Patient/parent resistance | ‘I think initially when we first made the change there was certainly some parents that would come in and they would kind of let the nurses know that they didn’t think that their MDIs worked, so they wanted to have a nebulised treatment, they could be quite forceful. And there were some that initially they were still trying to use the nebulised treatment at home...they felt that they were going down in their level of treatment with MDIs.’ **A/PH**  ‘Well, there'd be a backlash, you'd end up in a lot more arguments and fighting about it. Ultimately what you would have to do is you would have to—you'd say that you'd phase it in but I really think that you need to sort of just break it off. I think phasing it in won't work because I think you'll slide, so I think you just ultimately have to say, no, this is what we are doing now.’ **Y/RN** |
| Patient/parent empowerment | ‘It is easier on the parents, too. What we do is what they do. We don't take over. So they still feel in control, I guess.’ **E/RN**  ‘I think it's empowered parents to deal with children's asthma better at home. Now, that they have the ability to start the MDIs at home and get them started, I think that's giving them a better sense of how they can keep their child at home instead of coming into emergency all of the time.’ **A/RN** |
| Parental stress/anxiety | ‘Yes, there is an age difference for sure. I think the parents with the young children; they bring them in obviously...really early. They’re more anxious for sure.’ **E/PH** |
| Over treating | ‘[Patients will say] ‘...well, if I go to Emerg, they are just going to give me ten puffs, so I am just going to try ten puffs and see what happens.’ So, that is definitely a concern.’ **Y/PH**  ‘If we start using the MDIs in the hospital, parents will mimic that, as opposed to coming into the hospital with kids who are moderately severe to severe and keep the kids at home. So we may end up seeing some kids that are worse. And the parents are managing it, when they need to be in getting the steroids.**’ Y/PH** |
| Under treating | ‘But a lot of families also come in after not using it frequently if at all. Sometimes they've used it once and it's been all day.’ **Y/PH**  ‘I think a lot of times parents will come in and you kind of ask: Well, when is the last time they had their Ventolin? Oh, I didn't give any. So this would help them to maybe realize to give it in the early stages and, who knows, prevent them from having them come in at all.’ **Y/RN** |
| Severity issues | ‘To be honest, I find that of the patients labelled ‘emergent’ and ‘this is a severe patient’, the vast majority of those, in my opinion, would meet the criteria for ‘moderate’, or even ‘mild’’. **E/PH**  ‘I think whatever scoring system you use there is still some subjectivity to your scoring system, and that I don’t know. I don’t see how we can get around that**.’ Y/PH** |
| ‘Sitting still’ | ‘I don't think it [the MDI/spacer] would be very effective because we get a lot of children from, like, the two to five that aren't always very cooperative. And, like, with the inspiration, expiration, they would all get admitted.’ **Y/RN**  ‘I would think it would be much better for the kids because they are not going to be as stressed, because I'm telling you, they don't like the mask. You can hear a kid yelling down the hallway and it would be much better off for them in the long run and the parents would be much calmer probably versus putting that mask on.’ **Y/RN** |

**E** = Early adopter; **A** = adopting; **Y** = Yet to adopt; **RN** = nurse/respiratory therapist focus group/interview; **PH** = physician focus group/interview
